# Diagnostic Approaches in Myeloid Sarcoma

**DOI:** 10.3390/cimb47020111

**Published:** 2025-02-10

**Authors:** Elzbieta Patkowska, Agnieszka Krzywdzinska, Iwona Solarska, Magdalena Wojtas, Monika Prochorec-Sobieszek

**Affiliations:** 1 Department of Haematopoietic Stem Cell Transplantation, Institute of Hematology and Transfusion Medicine, 02-776 Warsaw, Poland; 2 Immunophenotyping Laboratory, Department of Hematological Diagnostics, Institute of Hematology and Transfusion Medicine, 02-776 Warsaw, Poland; akrzywdzinska@ihit.waw.pl; 3 Molecular Biology Laboratory, Department of Hematological Diagnostics, Institute of Hematology and Transfusion Medicine, 02-776 Warsaw, Poland; isolarska@ihit.waw.pl (I.S.); mwojtas@ihit.waw.pl (M.W.); 4 Pathomorphology Laboratory, Department of Hematological Diagnostics, Institute of Hematology and Transfusion Medicine, 02-776 Warsaw, Poland; monika.prochorec@interia.pl

**Keywords:** myeloid sarcoma, acute myeloid leukaemia, diagnostic approaches

## Abstract

Myeloid sarcoma (MS), or extramedullary acute myeloid leukaemia tumour (eAML), is a rare hematopoietic neoplasm. Recognised as a distinct entity within acute myeloid leukaemia (AML), MS presents significant diagnostic challenges due to its rarity, clinical heterogeneity, and variable immunophenotypic and genetic characteristics. The mechanisms by which leukaemic stem cells (LSCs) migrate to form solid tumours in extramedullary (EM) sites remain unclear. MS can occur de novo, precede AML, and manifest alongside AML relapse. It can also develop with myelodysplastic syndromes (MDSs) or myeloproliferative neoplasms (MPNs). MS frequently presents in organs such as the skin, lymph nodes, gastrointestinal (GI) tract, and central nervous system (CNS), often resulting in diverse clinical manifestations. Diagnosis relies on a comprehensive approach, including tissue biopsy, bone marrow (BM) evaluation, and advanced imaging modalities. Accurate diagnosis is crucial for risk stratification and treatment selection. Prognosis is influenced by several factors: MS’s anatomical location, timing of MS diagnosis, genetic profile, and possible treatment. This review emphasises the need for comprehensive diagnostic methods to better define individual MS characteristics and prognosis. It explores the role of novel targeted therapies in improving patient outcomes and further highlights the critical need for future multicentre data collection to optimise diagnostic and therapeutic approaches.

## 1. Introduction

Myeloid sarcoma (MS)—or its more precise term, extramedullary acute myeloid leukaemia (eAML) tumour—is a rare hematopoietic neoplasm. It originates from early-stage myelopoietic cells with maturation arrest at the blast phase in tissues other than the bone marrow (BM) [1]. MS, also referred to as chloroma, myeloblastoma, or granulocytic sarcoma, is recognised as a distinct AML entity, occurring in all subtypes of AML according to the World Health Organization (WHO) classification 2022, as well as the International Consensus Classification (ICC) 2022. However, MS is a heterogeneous disease rather than a homogeneous single entity. Knowledge of MS remains limited, mainly due to its rarity, clinical, immunophenotypic, cytogenetic/molecular characteristics heterogeneity, and inconsistent findings in reported studies. Factors predisposing to MS development are similar to those promoting AML and include occupational, environmental, lifestyle, and genetic factors [2]. However, the mechanisms by which leukaemic stem cells (LSCs) seed in tissues to form solid tumours remain poorly understood. Some studies suggest that the mobilisation and migration of LSCs from the BM to peripheral blood (PB), the spleen, and extramedullary (EM) sites may be influenced by several factors. These include the impaired adhesion of LSCs to the BM stroma due to altered expression of adhesion molecules, chemokine receptors/ligands, and integrins. Additional mechanisms, such as the activation of the Rat Sarcoma–Mitogen-Activated Protein Kinase/Extracellular signal-regulated Kinase (RAS-MAPK/ERK) pathway and deregulation in epithelial–mesenchymal transition (EMT) pathways further contribute to this process [3,4,5,6]. MS can occur at any stage of AML, such as several months before BM involvement, concomitantly with BM involvement at the time of AML diagnosis, or during AML relapse in either BM remission or non-remission. This includes AML relapse following chemotherapy or non-intensive treatment of AML, as well as relapse after allogeneic hematopoietic cell transplantation (allo-HCT) [7]. There is a lack of data on MS in patients who have undergone allo-HCT and are not in complete remission. MS can also coexist with myelodysplastic syndrome (MDS), myeloproliferative neoplasm (MPN), or myelodysplastic/myeloproliferative (MDS/MPN) syndrome [8]. The incidence of MS is estimated to range from approximately 2% to 18%, with a slight predominance in males and patients aged 46 to 59 years in the adult population [9,10,11,12,13]. MS occurs based on AML de novo in 18–87% of cases. Considering the type of underlying myeloproliferative neoplasm involved in the development of MS, it is observed in 20% of cases with MDS, 4% with chronic myelomonocytic leukaemia (CMML), and 2% with polycythaemia vera (PV) [14,15,16,17,18,19,20,21]. When MS occurs at a frequency of 12–20% in chronic myeloid leukaemia (CML), it indicates the myeloblastic phase of CML [22]. Rarely, MS may develop due to systemic mastocytosis or acute lymphoblastic leukaemia (ALL) [23,24]. The mutational landscape is variable, with frequencies of FMS-like tyrosine kinase 3 (*FLT3*), nucleophosmin *1* (*NPM1*), neuroblastoma RAS viral oncogene homologue (*NRAS*), isocitrate dehydrogenase 2 (*IDH2*), DNA methyltransferase 3a (*DNMT3A*), and ten-eleven translocation 2 (*TET2*) mutations similar to those observed in AML de novo [14,15,16]. MS can present as a single solid tumour or disseminated lesions. With or without intramedullary involvement, tissue and organ involvement cause various symptoms and highly variable clinical manifestations [17]. Organs most often involved include the skin, connective/soft tissues, lymph nodes, genitals, breast, gastrointestinal (GI) tract, peritoneum, bone, and central nervous system (CNS) [18,19,20,21]. Diagnosis is often challenging, and a comprehensive approach is necessary to ensure accurate diagnosis, risk stratification and treatment selection. The basis of diagnosis is histopathology with immunohistochemistry (IHC) of the tumour biopsy sample. The proper tissue triage and ancillary testing, including genetics and molecular profiling, are crucial. In addition to tumour biopsy, the broad diagnostic approach includes BM evaluation with immunophenotyping by flow cytometry (FCM), cytogenetic and molecular analysis, and appropriate imaging modalities. Of note, MS diagnosis in patients with coexisting AML or other hematologic malignancies is more straightforward than in de novo cases. A patient’s prognosis depends on several factors, including MS anatomical location, MS presentation timing, genetics, and treatment strategy. In the literature, the survival probability of MS patients is similar to the overall AML patient population [22,23]. However, a history of MDS or MPN has a negative impact on survival [23,24]. The prognosis of isolated MS must be more precisely characterised in the future [25]. This review provides an overview of the diagnostic approaches to MS, highlighting the crucial role of selecting a treatment for individual patients, potentially with novel therapy targeted to specific mutations.

## 2. Historical Perspective

Burns first described extramedullary tumours in 1811, and their relationship to AML was determined five decades later by Dock and Warthin [26,27]. Virchow used “weisses blut” and “leukaemia” terms in 1845 and 1847. King introduced the expression chloroma in 1853 due to the green colouration caused by myeloperoxidase (MPO) [28]. “Acute leukaemia” term was established in 1857. Later, Naegeli divided leukaemias into “myeloid” and “lymphocytic” in 1900 [29]. Dock and Warthin considered chloroma a more aggressive form of AML with metastases in 1902 [26]. The histologic term myeloblastoma was used in 1965 [30]. Finally, Rappaport introduced the currently preferred term MS of granulocytic origin in 1966 [31]. However, MS is implemented now for any AML or MDS tumour-related tumours; some authors suggest it should be named an eAML tumour [32].

## 3. Epidemiology

The epidemiology of MS is difficult to establish due to imprecise definitions, studies that included cases without histologic confirmation, and the fact that imaging modalities are not a standard for AML characteristics. Adult patients with MS typically range between 40 and 70 years of age, with males predominating [18,33,34]. The incidence of MS might be underestimated, and the frequency of MS with BM involvement at AML diagnosis ranged between 0.2 and 2.8%, and isolated MS 0.6 to 0.8% [35,36,37]. According to some authors, the rate of MS relapse following chemotherapy is not well determined in the literature. More data on relapses following allo-HCT are available; the frequency of isolated MS is 8–18%, and MS synchronous AML is 2–8% [24,35,36,38,39,40]. Approximately 70% of MS cases following allo-HCT are isolated forms without BM involvement [23,24,35,36,37,38,39,40,41,42]. The median time from allo-HCT, performed in CR1, to relapse as MS is 6 to 12 months [38,40]. Gingival infiltration, hematopoietic organ involvement (hepato- and splenomegaly, lymphadenopathy), leukaemia cutis (LC), and central nervous system involvement (CNSi) are most frequently observed in AML subtypes with myelomonocytic/monoblastic differentiation. Other locations include the soft tissue, bone, periosteum, orbit, paranasal sinuses, mediastinum, breast, gallbladder, GI and genitourinary tract [43]. The reported frequency of eAML varies significantly depending on the patient group studied.

## 4. Etiopathogenesis of EM AML

Factors predisposing to the development of MS are similar to those promoting AML. These include occupational, environmental, lifestyle, and genetic factors [2]. However, the mechanisms by which LSCs seed in tissues to form solid tumours remain poorly understood. Some authors suggest that the mobilisation and migration of LSCs from the BM to PB, the spleen, and EM sites may be influenced by several factors. These include impaired adhesion of LSCs to the BM niche due to altered expression and dysregulation of adhesion molecules, chemokine receptors/ligands, and integrins. Additional mechanisms, such as activation of the RAS-MAPK/ERK pathway, deregulation in EMT pathways, and pathogenic Nuclear Factor Erythroid 2 (*NEF2*) mutations, further contribute to this process [3].

The most common genetic abnormalities involved in the pathogenesis of de novo MS are t(8;21), inv(16), *KMT2A*r, *JAK2 V617F*, and in secondary cases, *KMT2A*r and *BCR::ABL1* [20]. Early studies identified the expression of neural cell adhesion molecule (NCAM, CD56) on leukaemic cells, commonly found in AML with t(8;21), as a potential risk factor for EM involvement [44,45,46,47]. It was hypothesised that high CD56 expression in the breast, testicular, ovarian, and gut tissue could have explained leukaemic cells’ affinity for these sites [45,48]. However, recent data report that the occurrence of CD56-positive blasts is similar in patients either with EM AML or without EM AML [35]. In AML with inv(16), disrupted regulation of core binding factor (CBF), which is a transcription factor involved in cell adhesion and recognition, seems to play a significant role in the pathogenesis of MS [49]. The aberrant expression of myeloid, B-cell, and T-cell antigens has been reported in MS [50]. T-cell antigens and CD56 expression on leukaemic cells were once considered relevant in cases involving the skin [51,52]. Elevated cutaneous lymphocyte antigen (CLA) expression has been described in a small group of patients with acute myelomonocytic leukaemia [53]. Leukaemic cell tropism to the skin may be explained by interactions of CLA with E-selectin on endothelial skin cells and interactions of intercellular adhesion molecule 1 (ICAM 1) with lymphocyte function-associated antigen (LFA-1) [54]. The expression of C-C motif chemokine ligand 3 (CCL3) on blasts and C-C motif chemokine receptor 5 (CCR5)/CCL3 interaction and the overexpression of C-X-C motif chemokine receptor 4 (CXCR4) and CXCR7 are speculated to be associated with blasts homing to cutaneous tissue [55,56]. Some similarities have been identified in the epigenetic microRNA profiles examined in skin biopsies, highlighting molecular-level connections between MS and blastic plasmacytoid dendritic cell neoplasm (BPDCN) and emphasising their relevance [57]. A study utilising single-cell RNA sequencing of BM and MS samples from LC cases identified a macrophage-like leukaemia subset enriched in MS and pre-existing in the BM, characterised by complement C1Q positivity. The findings demonstrate that C1Q expression, regulated by the transcription factor MAF BZIP, plays a pivotal role in enhancing the tissue infiltration capacity of leukaemic cells, underscoring its potential significance in the pathophysiology of the disease [58]. The metastasis-suppressor v-raf-1 murine leukaemia viral oncogene homologue 1 (*RAF*) kinase inhibitor protein (RKIP) loss has been reported in 50% eAML and only 14% AML without EM. RKIP loss and downstream sequelae of RAS-MAPK/ERK signalling may predispose to eAML. Recent research has highlighted significant dysregulation of the extracellular matrix (ECM)-receptor interaction and focal adhesion pathways in eAML [59]. ECM proteins have been implicated in a niche that supports cancer stem cells (CSCs) by providing structural and molecular support for their proliferation, self-renewal, and differentiation [60]. Notably, the upregulation of six collagen isoforms (COL), glycoproteins such as fibronectin (FN1), tenascin (TNC), thrombospondin (THBS2), and laminins (LAMA4/LAMB1) has been associated with the promotion of EMT, a process contributing to increased drug resistance [59]. Furthermore, the transcription factor TWIST1 has emerged as a pivotal regulator of apoptosis and EMT [61,62]. By interacting with adhesion molecules and other regulatory proteins, TWIST1 facilitates cancer cell invasion by disrupting epithelial–mesenchymal homeostasis. In eAML, TWIST1 expression is significantly elevated, further driving EMT-related processes and enhancing the invasive potential of leukaemic cells. These findings underscore the critical role of ECM dynamics and TWIST1 in the pathophysiology of eAML and their potential as therapeutic targets [59]. A summary of MS etiopathogenesis is presented in Table 1 [20,44,45,46,47,50,51,53,54,57,58,59,60,61,62,63].

## 5. Clinical Presentation

Symptoms of EM AML are determined by the site, tissue, and organ damage and the size of the infiltration, leading to a wide range of clinical manifestations [37,38]. The most common locations, independently of AML de novo or at relapse, include the soft/connective tissue (31–35%), skin/breast (11–46%), GI system (10–19%), reproductive organs (1–10%), bone (5–16%), head and neck (6–14%), and brain (4–11%) [18,24,34,36]. The skin, soft tissue, CNS, and GI are the most frequently involved sites in relapse following allo-HCT [38,39,64]. Skin infiltrates often present as single or disseminated erythematous nodules or papules [65]. The lesions are typically found on the lower extremities, upper extremities, trunk, and face [66]. LC sites may be localised in previous or current inflammation regions [66,67,68].

Gingival infiltration is characterised by gingival hypertrophy and regresses with chemotherapy [69]. Orbital involvement most frequently occurs in children and is associated with t(8;21) coexistence [70]. GI symptoms may include typhlitis, perirectal abscesses, and necrotising colitis caused by bowel wall infiltration with blasts. Dallan found that 94% of MS cases with *CBFβ-MYH11* or inv(16) present abdominal site involvement [71]. Cardiac abnormalities are related to electrolyte imbalances and infiltrations of the conduction system or vessel walls. Some studies have indicated that individuals of Asian descent may have a higher prevalence of cardiopulmonary or mediastinal involvement in certain conditions [36].

In the CNS, cerebrospinal fluid (CSF) is most commonly involved, potentially causing symptoms of leukaemic meningitis, less frequently brain, spinal cord, or cranial nerve infiltration. Suspected CNS involvement arises from rapidly progressing pathological neurological symptoms. Typical symptoms include headaches (40% of patients), mental status changes (50% of patients), walking difficulties (45% of patients), nausea and vomiting (12% of patients), and seizures (15% of patients) [72]. Cranial nerve palsies, particularly II, III, V, VI, and VII nerve, manifest as double vision (30% of patients), facial muscle weakness (25% of patients), hearing loss (20% of patients), trigeminal nerve neuropathy (12% of patients), and optic nerve neuropathy (2% of patients) [73,74]. Symptoms of increased intracranial pressure with persistent headaches, altered consciousness, and behavioural changes are often observed. CNS haemorrhage can lead to seizures, consciousness disturbances, headaches, or neurological deficits. Spinal cord involvement typically presents as diffuse back pain, lower limb weakness, paraesthesia, sensory disturbances, sphincter dysfunction, and asymmetry of reflexes [74]. MS localised in the brain presents as a solid tumour with mass effect and neurological deficits, whereas spinal canal localisation presents symptoms suggesting an extramedullary tumour [74,75]. Asymptomatic CNS involvement without pathological neurological symptoms is rare [74].

Approximately 70% of MS cases following allo-HCT are isolated forms without BM involvement [23,24,35,36,37,38,39,40,41,42]. The sites where leukaemic cells can survive chemotherapy and radiotherapy, resulting in relapse, are the CNS and reproductive organs, owing to inherent barriers and being like sanctuary sites [41,42]. Furthermore, CNS and other EM sites have noted a weak graft-versus-leukaemia (GvL) effect [76,77]. Beyond these hypotheses, identifying specific risk factors for MS in patients post-allo-HCT remains challenging. Although total body irradiation (TBI) used in conditioning can target the brain, testes, and ovaries more effectively than chemotherapy, it has not been shown to reduce the risk of post-allo-HCT relapse in these sites. No significant associations were found between the intensity of the conditioning regimen, graft source, and the presence or absence of acute or chronic graft-versus-host disease (GvHD) [78,79].

## 6. Diagnosis of MS

The cornerstone of diagnosing MS is obtaining tissue biopsy and performing a histologic examination with an IHC panel. According to WHO classification 2022, essential diagnostic criteria include effacement of tissue architecture by a mass composed of myeloid blasts, with or without maturing elements and positive immunophenotyping for granulocytic and/or monocytic markers. The proper tissue triage and ancillary testing, including immunophenotyping, genetics and molecular profiling, is crucial in tissue assessment. In addition to tumour biopsy, BM evaluation with immunophenotyping by FCM, cytogenetic and molecular analysis, and proper imaging modalities are necessary. Immunophenotyping by FCM complements IHC in defining the cell type and maturation degree at MS diagnosis and disease monitoring in further stages. Genetic and molecular abnormalities detection is pivotal to determining risk stratification and selecting an appropriate treatment option, especially in specific subtypes, including targetable mutations or acute promyelocytic leukaemia (APL). In selected cases, if the risk of biopsy is reasonable, a fine needle biopsy (FNB) or a radiologically guided core needle biopsy (CNB) of the tumour could be an alternative [80,81]. A comparison of different diagnostic techniques is presented in Table 2 [82].

## 7. Cytomorphology

MS lesions are classified by cell type and a broad myeloid differentiation and maturation spectrum. Morphologically, most cases consist of a diffuse and infiltrative population of myeloblasts, granulocytic cell components, monocytic cells, and rarely promyelocytes, erythroblasts, or megakaryoblasts. Cells are typically large, with abundant cytoplasm and large nuclei [83].

## 8. Histopathology

Fresh tissue is usually unavailable, although histopathological evaluation using IHC on formalin-fixed, paraffin-embedded (FFPE) performed on tissue samples is necessary to confirm MS diagnosis. A characteristic MS feature is the effacement of tissue architecture [80]. Cell morphology is assessed on slides using hematoxylin and eosin stain [84]. Although MS can present with various histological manifestations, it predominantly occurs in subtypes with myelomonocytic/monoblastic differentiation. Therefore, IHC is essential for detecting specific antigens and determining AML subtypes [35,85]. Frequently expressed antigens are MPO, CD33, CD13, CD68, and CD45 [18,33,86,87]. Tumours with an immature granulocytic profile express CD34 and KIT (CD117), whereas CD34 is negative in tumours with a more mature profile. Monoblasts and immature monocytic cells commonly express CD11b, CD11c, CD15, CD64, CD117, and lysozymes [18,81,88]. However, the expression of CD14 or CD34 is less frequent. More mature monocytic cells express CD14, CD68, CD163. Cells with megakaryocytic and erythroid differentiation express CD61 and glycophorin A. Markers of B cells, such as CD19, T cells, including CD4 and CD7, and NK cells with CD56, could be aberrantly expressed. Weak or only in a subset of cells, the expression of CD123, CD99, and TdT might be present [18,81]. Rarely do cases of aberrant cytokeratin expression as AE1/AE3 and CK8/18 occur [89,90]. Pileri evaluated the histopathological features of MS, finding diffuse growth patterns in extranodal sites and diffuse infiltration of sinuses or the paracortex in lymph nodes with residual lymphoid follicles involved. The most frequently reported immunohistochemical markers were CD68, often MPO, CD117, CD99, CD68/KP1, CD68/PGM1, less frequently CD34, CD56, and rarely CD61, CD30, CD4. High Ki-67/MIB1. Antigen values ranging from 50% to 95% were noted [18].

## 9. Flow Cytometry

The analysis of fresh tissue samples using FCM is recommended, and a comprehensive antigen panel should be conducted. FCM utilises a fluid stream to carry cells through a detector, examining various parameters of individual cells by evaluating the light they scatter or the photons emitted as they pass through a light source. Complementing tissue biopsy IHC, FCM allows for counting cells and immunophenotyping with cell differentiation at MS diagnosis and disease monitoring in further treatment phases. FCM after FNB or CNB could be the base for diagnosis in urgent situations or when a tissue biopsy is unavailable for IHC. In fact, FNA or CNB is rarely used and is usually insufficient to diagnose MS [91]. Cells usually detected in FCM are myeloblasts and/or monocytic cells. The pattern of surface antigen expression can differ among the different AML subtypes. Minimal panel of monoclonal antibodies should include CD34, CD117, CD13, CD15, CD33, HLA-DR, CD45, MPO for myeloblasts and CD64, CD14, CD4, CD36, CD13, CD15, CD11b, CD11c, CD56 for mature and immature monocytic cells identification. In rare erythroid or megakaryoblastic leukaemia cases, CD41, CD61 or CD71, CD105, and CD235a should be added. FCM may be particularly important in differentiating MS from extramedullary involvement in ALL and BPDCN cases. If possible, tissue biopsy IHC should always confirm the results of FNA or CNB immunophenotyping. Differences between immunophenotyping techniques IHC and FCM are presented in Table 3 [18,82,88,92,93,94].

## 10. Genetics

MS classification and treatment guidance require thorough genetic and molecular methodologies on FFPE tissue sections and BM. The recommended panel is similar to AML with BM involvement. However, the mutational spectrum of MS is not yet fully characterised, and insufficient data are available. Genetics includes fluorescence in situ hybridisation (FISH), conventional analysis by karyotype if possible, and molecular tests such as polymerase chain reaction (PCR) based and high-throughput sequencing with next-generation sequencing (NGS). The range of tests performed worldwide is highly varied, and the key factors affecting MS’s genetic profiling are patient’s age and geography differences. The following important point is the validation of a specific test for FFPE tissue to maintain reliability and accuracy. BM aspirate samples taken simultaneously should also be comprehensively tested. Even though they could contain no or rare leukaemic cells in the case of MS, significant gene mutations could still be detected in some patients. According to the studies, approximately 50–70% of MS patients present clonal cytogenetic abnormalities in FFPE. The most common is fusion genes, with a reported incidence of 57.1% in isolated MS [18,88,95]. Incidence of t(8;21) (q22;q22.1) *RUNX1::RUNX1T1* is reported of 28–38%, and of *KMT2A::MLLT3* of 19,2%, of inv(16) *CBFB::MYH11* is 10.3%, being similar in AML patients with and without MS in a meta-analysis [12,95]. A complex karyotype is reported in 4–39% of cases, more frequently in secondary MS [20]. Lysine methyltransferase 2A (*KMT2A*) gene rearrangement, monosomy 7, trisomy 4, trisomy 8, loss of chromosome Y, del(5q), and del(9q), monosomy 16, del(16q), del(20q), trisomy 11 are also reported [18,85,88,96,97]. The first most prevalent mutation in PCR-based tests is *FLT3-ITD*; the second is *NPM1* and *KMT2A*, although *DNMT3A* and *TET2* are also reported [12,32,85,98,99,100,101,102]. The pool prevalence of *FLT3* mutation of 17–30% and *NPM1* mutation of 17–50% in the meta-analysis is comparable to AML with BM involvement [12,101,102,103]. Furthermore, *KMT2A*, *DNMT3A TET2*, and stromal Antigen 2 (*STAG2*) mutations pool prevalence is 17.3%, 16.1%, 15.4%, and 12.8%, respectively, in meta-analysis [12]. Small studies reported the frequency of *IDH2* mutation at 20%, with a median variant allele frequency (VAF) of 40% and a frequency of *IDH1* mutation of 10.3% [97,103,104]. Rarely, MS cases with *Janus* kinase *2* (*JAK2*) mutation and *FIP1L1::PDGFRA* gene fusion with eosinophilia occur [20,88,97,101,102,105,106,107,108,109,110]. In several studies, quite a high frequency (30–85%) of gene mutations in the RAS pathway (*KRAS*, *NRAS*, *BRAF*, *PTPN11*, and *CBL*) were reported [96,97,102]. Gene fusions including *FUS::ERG*, *PICALM::MLLT10*, *ETV6::MECOM*, *NPM::MLF1*, as well as *TP53*, *RUNX1*, *ASXL1*, *WT1*, *SF3B1*, *SRSF2*, mutations are less frequent [96,97,102,103]. *BCR::ABL1* mutation, followed by *KMT2A*, was most prevalent in secondary MS. Molecular changes in MS and concurrent BM disease are consistent in approximately 70% of patients, suggesting that MS may originate from a standard hematopoietic stem cell or precursor. The relevance of cytogenetic and molecular mutations in isolated MS is not fully established yet and requires future larger studies. The frequency of selected mutations in MS is presented in Table 4 [18,96,98,101,102,103,104].

## 11. Imaging Tests

Imaging modalities play a pivotal role in characterising MS lesions and treatment monitoring. Initially, they help differentiate from other pathological changes, including abscesses or hematomas. The choice of modality depends on the MS anatomic location. Computed tomography (CT) is recommended for soft tissue infiltrations [111]. 18FDG positron emission tomography-computed tomography (18FDG-PET CT) is more effective than simple CT in detecting MS lesions in different localisations. It resulted in 77% sensitivity and 97% specificity. Magnetic resonance imaging (MRI) with or without gadolinium contrast is recommended for CNS evaluation with brain, spine, orbit tumours, and musculoskeletal lesions [83]. Of most importance, CNS involvement diagnostics should begin with imaging studies to exclude intracranial haemorrhage or the presence of a solid tumour with a mass effect. Subsequently, invasive procedures with lumbar punction or stereotactic brain biopsy are recommended [112]. In imaging tests, the MS lesions are usually nodules, soft tissue infiltrations, or disseminated processes resembling lymphoma. They are isodense compared to muscle in CT scans and isodense or hyperdense in T1- and T2-weighted MRI images [111,113,114,115]. Multiple anatomical EM locations were reported in 10–21% of MS patients [18,20,94,116,117]. Stölzel demonstrated the usefulness of PET-CT in determining the number, location, and size of infiltrations [118]. 18FDG-PET CT also benefits radiation therapy (RT) planning and monitoring treatment response [3,119].

## 12. Differential Diagnosis

Diagnosis of MS is often challenging, especially in isolated forms. Significantly, the aberrant antigenic expression can be misleadingly diagnosed correctly. Early studies reported misdiagnosis in 75–100% of MS de novo ases, frequently mistaken for lymphoma. Subsequent studies still reported misdiagnosis in 25–47% of cases [81,120,121,122]. Incorrect diagnoses included haematological neoplasms: ALL, non-Hodgkin lymphoma (NHL), Hodgkin lymphoma (HL), BPDCN, accumulation of mature hematopoietic cells in advanced stage of MPN (extramedullary haematopoiesis), and non-hematolymphoid tumours, including melanoma, histiocytic tumours, Ewing sarcoma, poorly differentiated carcinoma, and carcinoma metastases [43,123]. These diseases should be considered in the differential diagnosis, as well as benign ones with reactive MS-like tumours following granulocyte colony-stimulating factor (G-CSF), non-effacing extramedullary blastic proliferation [124]. In MS misdiagnosis, transformation to AML with BM involvement occurred after an average of 10.5 months [43]. This highlights the potential consequences of misdiagnosis and the urgent need to implement a comprehensive diagnostic approach.

## 13. Treatment of MS

**Figure 1:**
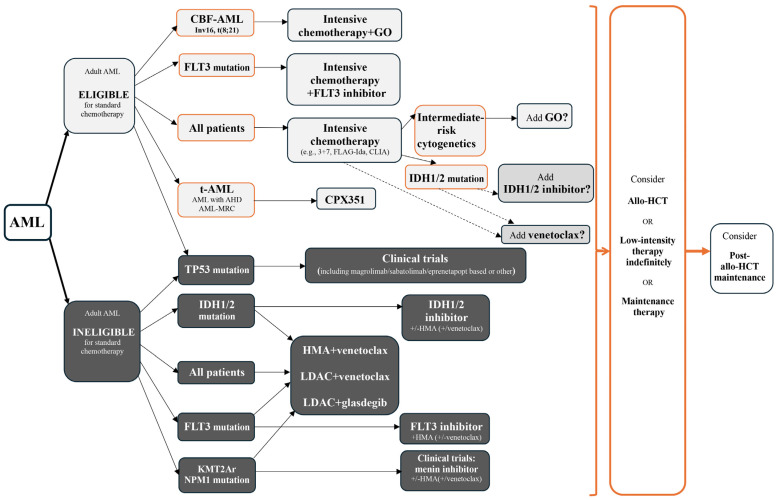
The frontline setting’s treatment paradigm for acute myeloid leukaemia based on ESMO 2020, ELN 2022, and NCCN version 1.2025 guidelines. AML, acute myeloid leukaemia; CBF, core-binding factor; FLT3 ITD, fms-related receptor tyrosine kinase internal tandem duplication; FLT3 TKD, fms-related receptor tyrosine kinase domain; IDH, isocitrate dehydrogenase; t-AML, therapy-related acute myeloid leukaemia; AML AHD, acute myeloid leukaemia with the antecedent haematologic disorder; AML-MRC, acute myeloid leukaemia with myelodysplasia related cytogenetic changes; TP53, tumour protein p53; KMT2Ar, lysine methyltransferase 2a rearrangements; NPM1, nucleophosmin 1; GO, gemtuzumab ozogamicin; CD33 ADC, CD33 antibody–drug conjugate; CPX351, liposomal daunorubicin and cytarabine; HMA, hypomethylating agent; LDAC, low-dose cytarabine; HCT, haematopoietic cell transplantation; ESMO, European Society for Medical Oncology; ELN, European LeukaemiaNet; NCCN, National Comprehensive Cancer Network. 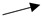 Standard option, 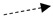 Alternative option.

**Figure 2:**
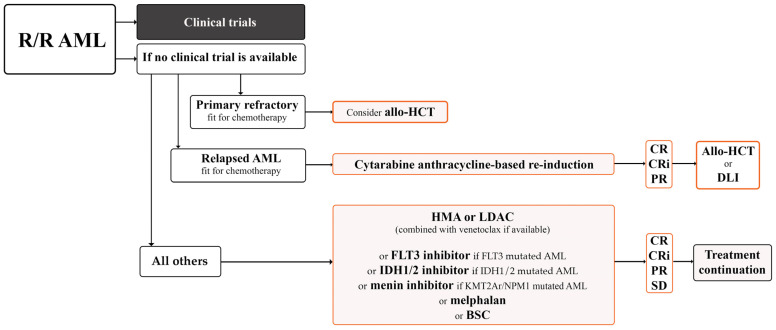
The salvage setting’s treatment paradigm for relapsed/refractory acute myeloid leukaemia based on ESMO 2020, ELN 2022, and NCCN version 1.2025 guidelines. R/R AML, relapsed/refractory acute myeloid leukaemia; FLT3, fms-related receptor tyrosine kinase; IDH, isocitrate dehydrogenase; KMT2Ar, lysine methyltransferase 2a rearrangements; NPM1, nucleophosmin 1; HMA, hypomethylating agent; LDAC, low-dose cytarabine; allo HCT, allogeneic haematopoietic cell transplantation; DLI, donor lymphocyte infusion; BSC, best supportive care; CR, complete remission; CRi, complete remission with incomplete haematological recovery; PR, partial remission; SD, stable disease; ESMO, European Society for Medical Oncology; ELN, European LeukemiaNet; NCCN, National Comprehensive Cancer Network.

MS is equivalent to AML, and it is recommended that it be treated with systemic chemotherapy and initiated as soon as possible after diagnosis [3]. However, due to the lack of prospective randomised studies, optimal treatment guidelines for patients with MS have yet to be established. Treatment recommendations come from retrospective analyses of small patient groups and case reports. Treatment selection primarily depends on the form: de novo vs. relapse of MS, type of infiltrate: isolated MS vs. BM involvement, cytogenetic/molecular profile, patient age, performance status (PS), and comorbidities [3]. Variable treatment options include systemic chemotherapy, novel targeted therapy, immunotherapy, individualised use of allo-HCT, and local treatment with RT, rarely with surgery [3]. Targeted therapies, with FLT3, IDH1/IDH2, Bcl-2 inhibitors, mennin inhibitors, and epigenetic mechanism-targeting drugs (hypomethylating agents [HMA], histone deacetylase inhibitors), also anatomically targeted drug CPX351 may become valuable adjuncts either to standard MS chemotherapy or lower-intensity regimens [125,126]. The efficacy of the standard lower-intensity treatment with HMA and venetoclax combination remains unclear; however, in recent studies, inferior outcomes in eAML were observed compared to those with AML without eAML [127,128]. The first-line treatment strategy for AML patients is presented in Figure 1 [129,130]. The indications for allo-HCT in patients with MS are generally consistent with those for AML, and it should be considered for all eligible patients, particularly those with high-risk disease [131]. RT is recommended for palliation of symptomatic vital structure compression in patients with isolated MS and inadequate response to systemic chemotherapy or isolated relapse after allo-HCT [3,132]. In the management of relapsed MS, therapeutic options extend beyond RT to include systemic salvage chemotherapy utilising agents not previously administered, HMA either as monotherapy or in combination with venetoclax, and, where feasible, an allo-HCT, also donor lymphocyte infusion (DLI) may be considered [32,133,134]. The treatment strategy for relapse/recurrent (R/R) AML patients is presented in Figure 2 [129,130].

## 14. Prognostic Implications

The prognosis for patients with MS is influenced by several factors, including the anatomical location of the lesion, the timing of its presentation, genetic/molecular mutations, and the treatment strategy. Extensive retrospective studies have failed to demonstrate a distinct prognostic difference between patients with MS and those with AML without MS or MS synchronous AML [19,35]. However, a history of MDS or MPN has been associated with poor survival outcomes [23,24]. Pre-transplant eAML or MS has not adversely affected allo-HCT outcomes [117]. Interestingly, in cases of relapse following allo-HCT, patients with isolated MS have demonstrated superior outcomes compared to those with AML without MS or MS synchronous AML as relapse [39]. These findings suggest that the clinical context of MS presentation and its relationship with other hematologic conditions may be critical in determining patient prognosis. Additionally, Sun reported that among patients who achieved their first complete remission (CR1) following induction chemotherapy, allo-HCT did not offer a significant advantage in 5-year overall survival (OS) compared to intensive chemotherapy alone. Conversely, in patients who did not achieve CR1, allo-HCT significantly improved 5-year OS. Furthermore, outcomes regarding OS, disease-free survival (DFS), and cumulative incidence of relapse (CIR) were comparable between HLA-matched and haploidentical HCT. Overall, allo-HCT was associated with better outcomes in MS patients within 36 months of disease onset, with haploidentical HCT representing a viable alternative for those without a matched donor [131]. However, after 36 months, the higher relapse rate after transplantation diminishes the survival advantage provided by the procedure [131].

## 15. Conclusions

The accurate diagnosis of MS requires a comprehensive diagnostic approach, incorporating conventional and advanced genetic methods. Histopathological evaluations of tissue samples, supported by IHC, are essential for diagnosis confirmation. In addition to IHC, genetic profiling and imaging modalities provide critical insight for risk stratification, tailored treatment selection, and effective disease monitoring. 18FDG-PET has proven highly valuable for evaluating disease extent and activity. The advent of diagnostic approaches and targeted therapies is significant for improving patient outcomes. Consequently, there is a need for well-designed studies, data collection, and diagnostic framework development to optimise diagnostics and treatment strategies in MS.

## Figures and Tables

**Table 1:** Etiopathogenesis of myeloid sarcoma.

Etiopathogenesis of Myeloid Sarcoma		
General factors predisposing to AML	Occupational exposures	Workers exposed to rubber, paint, embalming fluids, pesticides, ethylene oxide, petroleum, poultry, munitions, automobiles, nuclear power, plastics, and electrical wiring, as well as gasoline station attendants, beauticians, barbers, and cosmetologists.
	Environmental factors	Ionising radiation
	Lifestyle-related factors	Smoking, obesity
	Heritable genetic factors	*CEBPA*, *DDX41*, *RUNX1*, *ANKRD26*, *ETV6*, *GATA2*, *ELANE*, *HAX1*, *G6PC3*, *MPL*, *RBM8A*, *SBDS*, *SRP72*, trisomy 21, monosomy 7
	Clonal haematopoiesis	*DNMT3A*, *TET2*, *JAK2*, *ASXL1*, *TP53*, *GNAS*, *PPM1D*, *BCORL1*, *SF3B1*, *EZH2*
	Therapy-related AML	Cytotoxic therapy: alkylating agents, topoisomerase II inhibitors, nucleoside analogues, anti-tubulins Radiation therapy
Factors predisposing to MS	Genetics	t(8;21), inv(16); KMT2Ar, CBFB, monosomy7, del(5q), del(9q), loss of X, loss of Y, trisomy 4, trisomy 8
	Molecular abnormalities	(1) RAS pathway: *NRAS*, *KRAS*, *PTPN11*, *CBL*, *NF1*, *BRAF* (2) Activated signalling: *FLT3*, *BCR::ABL1*, *JAK2*, *KIT*, *SH2B3*, *CBL*, *CSF3R* (3) DNA methylation: *DNMT3A*, *KMT2A*, *TET2*, *IDH1*, *IDH2* (4) Cohesin complex: *STAG2*, *RAD21*, *SMC3* (5) RNA splicing: *SRSF2*, *SF3B1*, *U2AF1* (6) Transcription factors: *GATA2*, *RUNX1*, *CEBPA*, *ETV6*, *BCOR*, *BCORL1*, *STAT3* (7) Chromatin modification: *ASXL1*, *EZH2*, *SETD2* (8) Tumour suppressors: *TP53*, *WT1*, *NF1*, *PHF6* (9) Others: *NPM1*, *SETBP1* Most patients harbour multiple mutations (>1), with the most frequently observed being: *NPM1*, *NRAS*, *DNMT3A*
	Cells morphology	Monocytic component
	Aberrant antigen expression	Aberrant expression of myeloid cell, B-cell: CD19, T-cell: CD4, CD7, NK-cell: CD56 antigens
	Pathogenic pathways	BONE MARROW AND EXTRAMEDULLARY NICHE Regulatory abnormalities: (1) Loss of RAF kinase inhibitor protein and activation of RAS-MAPK/ERK pathway (2) Deregulation in the EMT pathway, altered ECM–receptor interactions and local adhesion pathways, formation of a CsC-supportive niche Contributors to EMT and invasiveness: (1) ECM proteins, the upregulation of six collagen isoforms: FN1, TNC, THBS2, and LAMA/LAMB1, the promotion of EMT and drug resistance (2) The role of transcription factor TWIST1 in EMT and apoptosis: the interactions with adhesion molecules, the disruption of the epithelial–mesenchymal balance, the enhancement of leukemic cell invasion, the promotion of EMT, and the increase in overall invasiveness STEM CELL MIGRATION (1) Altered expression and dysregulation of membrane adhesion molecules, chemokine receptors/ligands, and integrins (2) Impaired adhesion of LSCs to BM niches, the mobilisation and migration of LSCs into PB, spleen, and EM sites (3) Overexpression of CXCR4, CCR5, CX3CR1, CXCR7, CCR2 (4) Interactions between CLA and E-selectin on skin endothelial cells; ICAM-1 with LFA-1; CCL3 with CCR5 on blasts (5) Expression of PD-L1 (6) Activation of the RAS-MAPK/ERK pathway (7) Pathogenic *NEF2* mutations (8) Presence of macrophage-like leukaemia subset with complement C1Q positivity
	Epigenetic/microRNA profiles	Some cases shared features between MS and BPDCN

AML, acute myeloid leukaemia; MS, myeloid sarcoma; RAS, viral (v-ras) oncogene homologue; RAF v-raf-1, murine leukaemia viral oncogene homologue 1; RAS-MAPK/ERK, rat sarcoma-mitogen-activated protein kinase/extracellular signal-regulated kinase; EMT, epithelialmesenchymal transition; ECM, extracellular matrix; CSC, cancer stem cells; LSCs, leukaemia stem cells; BM, bone marrow; PB, peripheral blood; CXCR4, C-X-C motif chemokine receptor 4; CCR5, C-C motif chemokine receptor 5; CLA, cutaneous lymphocyte antigen; ICAM-1, intercellular adhesion molecule 1; LFA-1, lymphocyte function-associated antigen 1.

**Table 2:** Comparison of tissue biopsy and body fluid diagnostic methods for myeloid sarcoma diagnosis.

	Surgical (Excisional) Tissue Biopsy	Fine Needle Biopsy	Core Biopsy	Body Fluids (e.g., Cerebrospinal Fluid, Pleural Effusion)
Tissue architecture	Undisturbed, broad heterogeneity	Disrupted	Locally undisturbed, reduced heterogeneity	Not applicable
Morphology	Low-power architecture and high-power cytomorphology	Detailed cellular morphology	Low-power architecture limited by biopsy size	Detailed cellular morphology
Procedure invasiveness	Surgical excision procedure The most invasive procedure	The least invasive procedure The lowest risk of complications Image guidance may be necessary	A minimally invasive procedure The possibility of performing percutaneously or endoscopically. Image guidance may be necessary.	Usually less invasive than direct tissue biopsy
Limitations	Accessibility may be challenging depending on the anatomical location (e.g., brain, retroperitoneum, mediastinum).	Inappropriate for cases with rare malignant cells or structural characteristics that limit aspiration (e.g., fibrosis, necrosis).	Inappropriate for cases requiring analysis of extensive heterogeneity or scarce malignant cells.	Often relatively low in cellularity, mainly when obtained from small-volume or relatively sterile sites. Limited effectiveness of specific techniques, e.g., cell block preparation.
Most appropriate for diagnostics	Cell block preparation. Scarce malignant cells and/or diagnostic features depending on low-power architectural distortion.	Homogeneous populations of readily identifiable (immunophenotypically aberrant) cells	Aberrant cell populations homogeneous or mixed, or displaying distinct architectural patterns. Biopsy of deep or relatively inaccessible lesions that are not readily amenable to surgical resection.	Assessment of cells in cerebrospinal fluid, pleural, pericardial, peritoneal effusions, bronchoalveolar lavage, and vitreous fluid.
Morphologic examination methods	Cells collected into a tissue block and embedded in paraffin and immunophenotyping via IHC.	Cells smeared on glass slides and stained or collected into a tissue block and embedded in paraffin and immunophenotyping via IHC	Cells smeared on glass slides and stained or collected into a tissue block and embedded in paraffin and immunophenotyping via IHC	The workup used techniques similar to those used in FNA samples: stained smears for cytomorphology and collected tissue fragments for embedding into a cell block.
Type of obtained cells	Fixed cells	Viable and intact, disaggregated cells	A reasonable number of viable cells suitable for morphology assessment.	Viable cells
Diagnostics methods	Immunophenotyping by IHC, FISH, NGS	Immunophenotyping by flow cytometry, conventional karyotyping, FISH and a wide range of molecular genetic studies Cultures	Immunophenotyping by IHC and flow cytometry, conventional karyotyping, FISH and a wide range of molecular genetic studies Cultures	Immunophenotyping by flow cytometry, conventional karyotyping, FISH and a wide range of molecular genetic studies Cultures
Characteristics	The highest potential diagnostic yield.	The intermediate potential diagnostic yield.	The intermediate potential diagnostic yield. It could be combined with FNA, similar to the combination of bone marrow aspirate and core biopsy.	The intermediate potential diagnostic yield. Depending on the site, e.g., cerebrospinal fluid, pleural or peritoneal effusions, or bronchoalveolar lavage.

IHC, immunohistochemistry; FISH, fluorescence in situ hybridisation; NGS, next-generation sequencing; FNA, fine needle aspiration.

**Table 3:** Comparison of diagnostic immunophenotypic methods for myeloid sarcoma diagnosis. MS, myeloid sarcoma.

	Flow Cytometry	Immunohistochemistry
Markers number analysed	Many (typically 8–12 in the majority of clinical laboratories)	One (occasionally two)
Spectrum of measurable intensity	Broad	Limited
Category of information collected	Quantitative	Qualitative
Processing duration	Fast (under 1 h)	More time-consuming (several hours)
Sample	Undamaged, disaggregated cells	Cryopreserved tissue or formalin-fixed, paraffin-embedded tissue samples
Correlation with cell morphology	Indirect	Direct
Most appropriate for	Samples of non-cohesive live cells require analysis of multiple markers per cell—no critical correlation with morphology.	Paraffin-embedded samples or populations not suited to disaggregation. Limited markers per cell analysed—uncommon populations of cells with morphological abnormalities.
Characteristics	Counting cells and immunophenotyping with cell differentiation	The effacement of tissue architecture. Pleomorphic infiltrate of early-stage myelopoietic cells with maturation arrest at the blast phase. Variable sizes of cells.
Antigens expressed	Myeloblasts: CD34, CD117, CD13, CD15, CD33, HLA-DR, CD45, MPO Mature and immature monocytic cells: CD64, CD14, CD4, CD36, CD13, CD15, CD11b, CD11c, CD56 Megakaryoblastic or erythroid differentiation: CD41, CD61 or CD71, CD105, and CD235a	Frequently: MPO, CD33, CD13, CD68, and CD45 An immature granulocytic profile: CD34 and KIT (CD117) Monoblasts and immature monocytic cells: CD11b, CD11c, CD15, CD64, CD117 and lysozyme Less frequently: CD14 or CD34 More mature monocytic cell: CD14, CD68, CD163 Megakaryoblastic and erythroid differentiation: CD61 and glycophorin A. Exceptionally aberrantly expressed: markers of B-cells: CD19, T-cells: CD4, CD7 and NK-cells: CD56 Weak or only in a subset of cells: CD123, CD99, and TdT Rarely: aberrant cytokeratin expression as AE1/AE3 and CK8/18 Non-specific but highly sensitive for MS: lysozyme, CD68, CD43

**Table 4:** Mutations frequency in patients with myeloid sarcoma.

ELN 2022 Risk Category by Genetics at AML Diagnosis	Mutated Genes and Genetic Alterations	Rates (%) in MS Patients
Favourable	RUNX1::RUNX1T1	2–23
	CBFB::MYH11	9–17
	NPM1	15–54
Intermediate	FLT3-ITD	6–15
	MLL rearrangement	7–11
Adverse	TP53	8–22
	RUNX1	7–11
	Monosomy 7	8–11
	Del(5q)	5–8
Cytogenetic and/or molecular abnormalities not classified by ELN as favourable or adverse	NRAS	11–31
	KRAS	11–15
	IDH1	15
	IDH2	11–31
	DNMT3A	8–28
	TET2	17–22
	FLT3-TKD	17
	PTPN11	11–15
	KIT	14–15
	CBL	11
	Trisomy 8	11–15

ELN, European LeukemiaNet; AML, acute myeloid leukaemia; MS, myeloid sarcoma.
